# Public transit and methadone – Spatial analyses of opioid treatment program access in Greater Boston, 2020–2022

**DOI:** 10.1016/j.pmedr.2025.103317

**Published:** 2025-11-20

**Authors:** Max R. O'Reilly, Thomas J. Stopka, Ric Bayly, Olivia Lewis, Shikhar Shrestha, Jack Cordes, Alexander Y. Walley, Sumeeta Srinivasan

**Affiliations:** a Department of Public Health and Community Medicine, Tufts University School of Medicine, 136 Harrison Ave, Boston, MA 02111, USA; b Department of Urban and Environmental Policy and Planning, Tufts University, 503 Boston Avenue, Medford, MA 02155, USA; c Boston University Chobanian & Avedisian School of Medicine, Boston Medical Center, 72 E Concord St., Boston, MA 02118, USA; d Massachusetts Department of Public Health Bureau of Substance Addiction Services, 250 Washington St. #3, Boston, MA 02108, USA

## Abstract

**Objective:**

Methadone, an FDA-approved medication, can effectively treat opioid use disorder (OUD). However, long travel times to opioid treatment programs (OTPs) commonly present barriers to initiating and continuing methadone treatment. We utilized geographic information systems to identify areas with unmet OTP access needs via public transportation and walking in Boston, Massachusetts.

**Methods:**

We created public transit-time and walk-time catchment areas around OTPs. We assessed spatial overlap between the catchment areas and locations of opioid-related overdose decedent residences from 2020 to 2022 in Boston, and inequities in OTP access by decedent race/ethnicity using logistic regression.

**Results:**

In Greater Boston, 50.1% of fatal opioid-related overdose decedent residences were within 30 min of an OTP via public transit at the time of death. In Boston, 80.4% of fatal opioid-related overdose decedent residences were within 30-min transit times of an OTP. In the early morning, access to OTPs on public transit is limited. Differences in OTP access based on decedent race/ethnicity were inconsistent.

**Conclusion:**

Many areas in Greater Boston have unmet OTP access needs on public transit. Our results can inform decision-making to improve OTP access in Boston, such as increased early-morning transit service, expanding access to methadone beyond existing OTPs, or broadening non-emergent medical transit.

## Highlights

• Access to opioid treatment programs within 30 min on public transit is limited
• In suburbs, access to opioid treatment programs via public transit and walking is nearly identical
• Inequities in access based on decedent race/ethnicity are not consistent
• Long public transit times are a barrier to care for patients without access to a car

## 1 Introduction

The opioid overdose epidemic is one of the largest American public health crises of the 21st century. Between 2002 and 2022, opioid-related overdose deaths nearly quadrupled; an estimated 54,743 opioid-related overdose deaths occurred in the US in 2024 (National Center for Health Statistics, 2025). Between 6.7 and 7.6 million people were estimated to be living with opioid use disorder (OUD) nationwide in 2019 (Keyes et al., 2022). However, a minority of those with OUD receive treatment, with estimates ranging from 8% (Romo et al., 2018) to 25% (Dowell et al., 2024), a figure that is lower among uninsured and communities of color (Wu et al., 2016). In 2023, the overdose-related death rate in Massachusetts was 30.2 deaths per 100,000 population (Massachusetts Department of Public Health, 2024).

To treat OUD and mitigate the risk of opioid-related overdose deaths, medications for opioid use disorder such as methadone are first line treatment (National Academies of Sciences, 2019). Methadone treatment is highly effective in preventing detrimental OUD-related outcomes, decreasing opioid-related overdose deaths by 59% during the 12-month period following a nonfatal opioid-related overdose (Larochelle et al., 2018) (Connery, 2015). Yet, methadone is accessible almost exclusively via opioid treatment programs (OTPs) (Pasman et al., 2022). Among myriad barriers to accessing methadone treatment, travel times can be considerable, as most patients must travel to an OTP in-person every day to receive treatment (Livingston et al., 2022). Prior research found that patients who traveled less than one mile to an OTP to receive methadone were 50% more likely to continue treatment compared to those travelling longer distances, lowering their risk for subsequent overdoses (Larochelle et al., 2018) (Beardsley et al., 2003), demonstrating the important role that geographic access to OTPs plays in methadone treatment retention.

Other studies have examined geographic access to OTPs and harm reduction services based on Euclidean distance (as-the-crow-flies), and travel by car (Cantor et al., 2021; Davidson et al., 2022; Mitchell et al., 2022; Parbs et al., 2024), but few have focused on treatment access via travel on public transportation (Bachhuber et al., 2025; Drake et al., 2020; Kim et al., 2024; Yücel et al., 2023). Car ownership is low in the City of Boston, and likely to be lower among those living with OUD, who are more likely to have a lower socioeconomic status (Albright et al., 2023). Thus, many patients in Boston seeking methadone treatment may utilize public transportation to access care, making geographic access to OTPs on public transportation a vital predictor of methadone treatment initiation and retention.

In this study, we analyzed geographic access to OTPs in Boston via public transportation and walking to identify areas with low or inequitable access to methadone treatment. These results can help guide preventive medicine-based decision-making, informing placement of future OTPs in locations served by public transit, or expansion of access to methadone beyond OTPs (e.g., mobile clinics), which can improve methadone treatment initiation and continuity, reducing opioid-related overdose mortality.

## 2 Methods

### 2.1 Data

We compiled fatal opioid-related overdose data from the Massachusetts Registry of Vital Records and Statistics (RVRS) and OTP location data from the Bureau of Substance Addiction Services (BSAS). OTP address locations from BSAS included all operating OTPs as of November 2023. As a proxy for individuals with OUD who may benefit from OTP services, we geocoded and mapped the residential addresses of opioid-related overdose decedents in Massachusetts from 2020 to 2022. We geo-masked decedent residences using bimodal Gaussian jittering to protect the anonymity of decedents and their families. Decedent residences, at the time of fatal overdose, represent a useful proxy for a pool of potential OTP patients because they indicate areas where there is a local burden of opioid-related overdose, which reflects geographic patterns of OUD (Pustz et al., 2022). Fatal opioid-related overdose decedent residences have been utilized as patient proxies in prior studies of geographic access to OTPs (Cao et al., 2019; Kim et al., 2024; Pustz et al., 2022).

We conducted analyses focused on two regions. First, Greater Boston, including all municipalities (*n* = 49) that receive MBTA rapid-transit train and bus service, consisting of a population of 2,557,317 (2020 Census). In addition, we replicated our analysis within the City of Boston, excluding surrounding municipalities, segmented by neighborhoods (*n* = 23), with a total population of 675,647 (2020 Census).

We aimed to model a hypothetical OTP patient travelling to receive methadone treatment on public transportation from their home. For transit-related travel, we used General Transit Feed Specification (GTFS) data from the MBTA which contained information on routes, stops, and schedules. To account for walking to public transportation stops, we used 2020 Census TIGER street data for Massachusetts.

### 2.2 Measures

**Supplementary Fig. 1:**
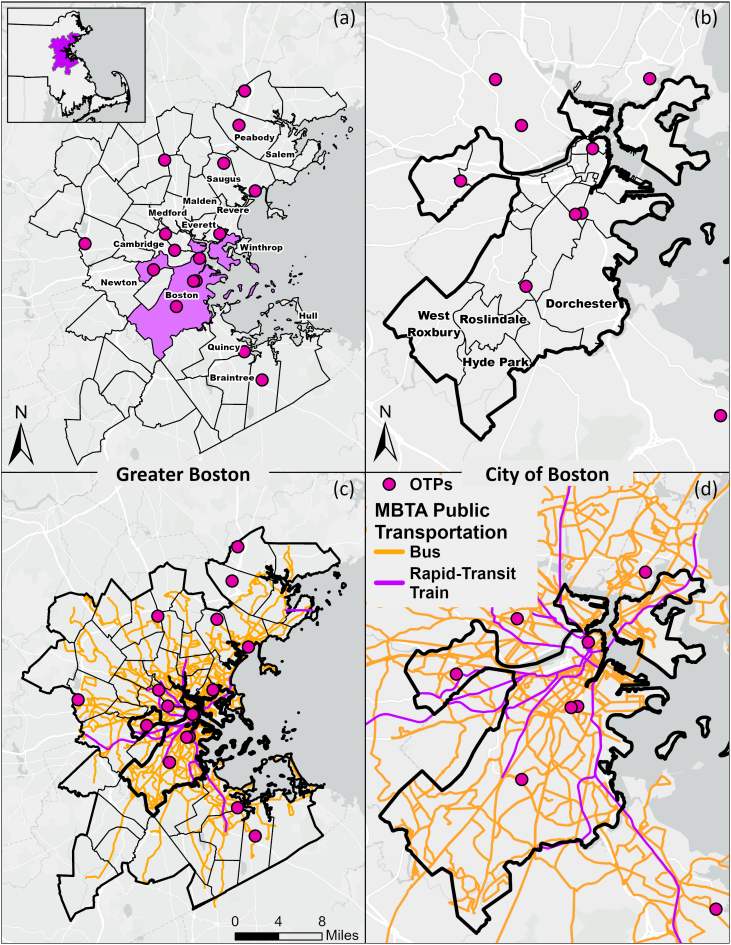
Highlighted municipalities in Greater Boston (a) and the City of Boston (b). Locations of all OTPs and MBTA routes bus and rapid-transit train routes in Greater Boston (c) and the City of Boston (d) (2022). Map lines delineate study areas and do not necessarily depict accepted national boundaries.

We used geographic information systems (GIS) to create a model that simulated a patient travelling on public transportation. Our model used GTFS and street data to simulate the walk to a public transit stop, the public transit trip, and the walk from the stop to an OTP. This model was similar to the public transportation option for directions on cell phone maps. Bus and train routes are shown in Supplementary Fig. 1; we excluded routes that included the commuter rail or ferry. The model generated an area that contained all residential locations within 30 min of an OTP on public transit, a catchment area referred to as a “service area polygon.” Any residences that fell outside the service area had travel times longer than 30 min one-way by public transit to an OTP, which constituted an undue travel burden to the patient (Del Conte et al., 2022).

Prior research on public transportation-based access has argued that it is inherently dynamic due to wait times in between buses/trains, traffic, and schedule changes (Farber et al., 2014). To account for these factors, we calculated service areas for every 3-min interval between 8:00 am and 8:30 am on a weekday. We utilized the smallest (i.e., smallest geographic area covered) of the service areas generated within the 30-min window, representing a conservative estimate of public transit accessibility that aimed to consider obstacles faced when taking public transit. We ran sensitivity analyses in the early morning (5:00–5:30 am, 5:30–6:00 am, … 7:30–8:00 am), when many patients receive treatment (Frank, 2021). We also generated 30-min walk-time service areas to compare accessibility to OTPs by solely walking versus using public transit and used a mean walking speed of 3 mph (Farber et al., 2014).

To calculate accessibility to OTPs using public transportation, we aggregated counts of decedent residences within walk and public transit service areas. We compared the two to determine which patients within 30 min of an OTP by public transit could have also arrived at an OTP by walking for 30 min or less.

### 2.3 Statistical analysis

To assess potential inequities in access to OTPs by decedent race and ethnicity, we used the race and ethnicity variable in the opioid-related overdose decedent dataset. The dataset contained four categories for race and ethnicity: non-Hispanic White, non-Hispanic Black, Hispanic, and other race and ethnicity. We aggregated 2020 Census Block Group data on race and ethnicity by municipality and neighborhood to bolster our understanding of equitable access to OTPs.

We constructed two binomial logistic regression models. The first, a single-level model, assessed whether a decedent's residence within 30-min of an OTP on public transit (yes/no) was associated with the decedent's race and ethnicity. The second model, a hierarchical logistic regression model, assessed whether the decedent's residence within 30 min of an OTP (yes/no) was associated with racial and ethnic composition of the municipality of the decedent's residence address and the race and ethnicity of the decedent. The second model utilized a robust covariance matrix to account for the clustering of decedent residences within the same municipality/neighborhood.

Analyses were conducted using ArcGIS Pro 3.2.3 (Esri, Redlands, CA), Python v3.10 (Beaverton, OR), and RStudio v2025.05.1 + 513 (Posit, Boston, MA). Our study protocol was reviewed, approved, and determined to be non-human subjects research by the Institutional Review Board of Tufts Health Sciences and the Massachusetts Department of Public Health.

## 3 Results

### 3.1 Geocoding

We achieved a 96% match rate for geocoded decedent residence addresses, resulting in a sample that included 2073 decedents, 711 of which were in the City of Boston, from the years 2020–2022. Demographic information on decedents is described in Supplementary Table 1. We successfully geocoded 100% of OTP locations (*n* = 16).

### 3.2 Transit time vs. walk time

**Fig. 1:**
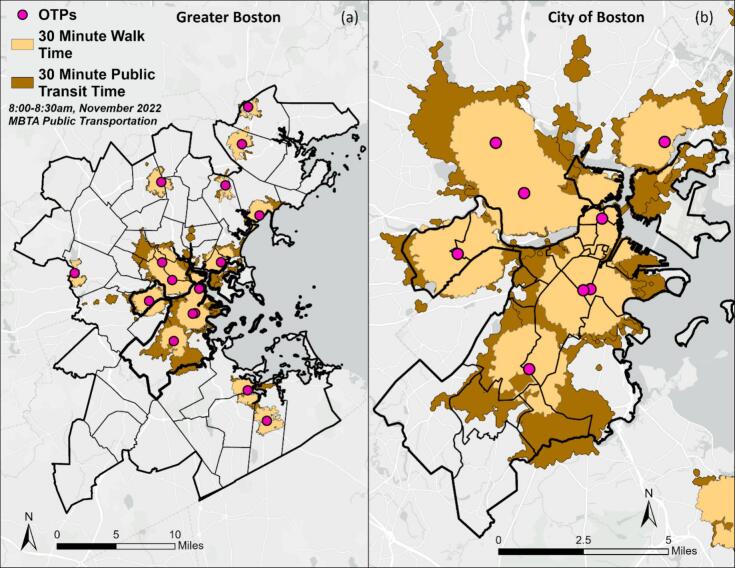
Service areas for travel to OTPs (pink points) by public transportation (brown) and walking (yellow). Brown areas represent the additional area covered by public transportation when compared to solely walking. Greater Boston (a) and the City of Boston (b), 2022. (For interpretation of the references to color in this figure legend, the reader is referred to the web version of this article.)

Across Greater Boston, 1038 of 2073 decedents (50.1%) resided within 30 min of an OTP on public transportation at the time of their death (Supplementary Table 2). Among decedents living within 30 min of an OTP via public transit, 67.4% (699 of 1038) were within a 30-min walk-time to an OTP, showing higher coverage of public transportation compared to walking. Public transportation better served the City of Boston and neighboring municipalities compared to suburban communities. In suburban areas, some of the boundaries of walk-time and transit-time service areas were identical, indicating no improvement in geographic access to OTPs using public transportation when compared to walking in those areas (Fig. 1).

### 3.3 Access to OTPs by public transportation

**Fig. 2:**
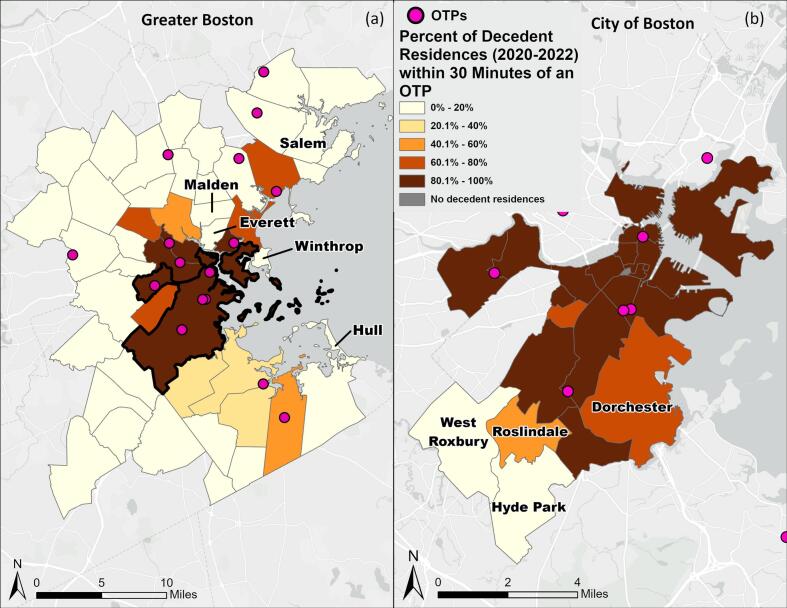
The percent of fatal opioid-related overdose decedent residences 2020–2022 within 30 min of an OTP on public transportation in each municipality of Greater Boston (a), and neighborhoods within the City of Boston (b).

We observed substantial variation in access to OTPs on public transportation across municipalities in Greater Boston. In 26 of 49 municipalities in Greater Boston, no decedent residences were within 30 min of an OTP by public transit, and an additional 14 municipalities had less than 50% of decedent residences within 30 min of an OTP on public transit (Fig. 2a). Malden, Everett, Winthrop, Salem, and Hull are all of note, with many fatal opioid-related decedent residences, but fewer than 10% of those residences within 30 min of an OTP by public transit (Fig. 2a, Supplementary Fig. 1).

In the City of Boston, 80.4% of decedents resided within 30 min of an OTP via public transportation (Supplementary Table 3). About 10% of decedent residences in the neighborhoods of West Roxbury and Hyde Park were within 30 min of an OTP via public transit (Supplementary Fig. 1). In Roslindale and Dorchester, about two-thirds of decedent residences were within 30 min of an OTP (Fig. 2b).

### 3.4 Access to OTPs by race and ethnicity

#### 3.4.1 Access to OTPs by race and ethnicity – Greater Boston

**Fig. 3:**
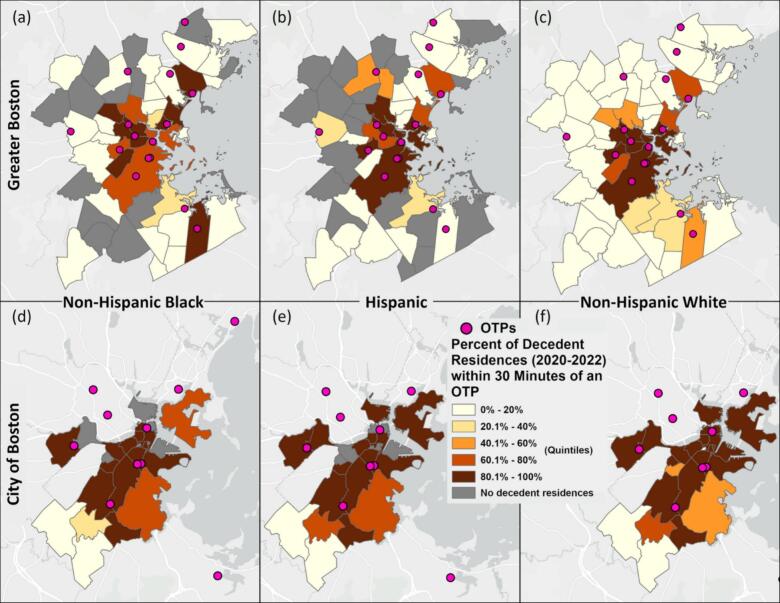
The percent of fatal opioid-related overdose decedent residences within 30 min of an OTP in Greater Boston (a-c) and in the City of Boston (d-f) for non-Hispanic Black (a, d) Hispanic (b, e), and non-Hispanic white decedents (c, f), 2020–2022.

In Greater Boston, non-Hispanic Black (OR = 3.22, 95% CI = (2.53, 4.12)) and Hispanic (OR = 2.93, 95% CI = (2.26, 3.82)) decedents had improved access to OTPs when compared to non-Hispanic White decedents. A 1% increase in proportion of non-Hispanic White individuals in a municipality was associated with 0.95 (0.91, 0.99) decreased odds that a decedent lived within 30-min of an OTP on public transportation (Table 1). This was evident in municipalities such as Cambridge, Everett, Medford, and Revere, where higher proportion of non-Hispanic Black and Hispanic decedents were within 30 min of an OTP on public transit when compared to non-Hispanic White decedents (Fig. 3a-c, Supplementary Fig. 1a-b). However, in some municipalities, including Braintree, Newton, Peabody, Quincy, and Saugus, a higher proportion of non-Hispanic White decedents resided within 30 min of OTPs by public transportation compared to decedents who were non-Hispanic Black, Hispanic, or another race and ethnicity. (Fig. 3a-c, Supplementary Fig. 1a-b).

**Table 1:** Associations between the race and ethnicity of fatal opioid-related decedents (model 1), the racial/ethnic composition of the town in which they resided (model 2), and their residences within 30 min of an OTP (Y/N) and local public transportation, Greater Boston and the City of Boston 2020–2022.

	*Model 1: Single Level*			*Model 2: Multilevel*	
	*Greater Boston*	*City of Boston*		*Greater Boston*	*City of Boston*
Indicator	OR (95% CI)	OR (95% CI)	Indicator	OR (95% CI)	OR (95% CI)
*Individual Decedent Level*			*Individual Decedent Level*		
*White non-Hispanic*	Ref	Ref	*White non-Hispanic*	Ref	Ref
*Black non-Hispanic*	3.22 (2.53, 4.12)	0.85 (0.55, 1.31)	*Black non-Hispanic*	2.00 (1.34, 2.97)	1.19 (0.77, 1.82)
*Hispanic*	2.93 (2.26, 3.82)	1.01 (0.62, 1.67)	*Hispanic*	1.91 (1.35, 2.69)	1.30 (0.81, 2.09)
*Other*	1.20 (0.59, 2.40)	0.45 (0.15, 1.50)	*Other*	1.05 (0.59, 1.89)	0.53 (0.14, 2.03)
*Municipality Level*			*Municipality Level*		
	–	–	*Proportion non-Hispanic White (municipality)*	0.95 (0.91, 0.99)	1.02 (0.98, 1.06)

#### 3.4.2 Access to OTPs by race and ethnicity – City of Boston

In the City of Boston, as compared to Greater Boston, access to OTPs by public transportation was better for decedents of all races and ethnicities, but low in some neighborhoods with more people of color (Fig. 3d-f). Hyde Park, Roslindale, and Dorchester had more non-Hispanic Black and Hispanic residents than the rest of Boston's neighborhoods, and lower OTP access: 6.3%, 57.9%, and 62.4% of decedents within 30 min of an OTP on public transit, respectively (Supplementary Table 3, [27]). There were no statistically significant relationships between a decedent's race and ethnicity and the odds of accessing an OTP within 30 min on public transit (Table 1).

### 3.5 Sensitivity analyses

Access to OTPs by public transit varied considerably based on the time of day that a hypothetical patient was travelling. Access to OTPs was considerably lower in the early morning when compared to peak travel hours (8:00–8:30 am) (Table 2).

**Table 2:** Percent of fatal opioid-related decedent residences in Greater Boston and the City of Boston (2020−2022) within 30 min of an opioid treatment program by travel on public transportation for different departure windows.

Percent of Decedent Residences Within 30 Minutes of an OTP by Public Transportation		
Departure Window	Greater Boston%	City of Boston%
5:00–5:30a	39.0	59.4
5:30–6:00a	47.6	78.3
6:00–6:30a	53.6	88.9
6:30–7:00a	52.6	88.7
7:00–7:30a	51.4	84.5
7:30–8:00a	48.9	76.5
8:00–8:30a	50.1	80.5

## 4 Discussion

We analyzed access to OTPs in Boston with the intent of highlighting areas with unmet OTP access needs via travel on MBTA public transportation. We found that half of fatal opioid-related overdose decedent residences in Greater Boston had greater than 30-min transit and walk time access to OTPs, with high unmet demand specifically in Everett, Malden, Winthrop, Salem, and Hull. Even within the City of Boston, many decedent residences in West Roxbury, Hyde Park, Roslindale, and Dorchester were not within 30 min of an OTP by walking or public transit. Proximal geographic access is associated with considerably higher treatment retention rates given the requirement for frequent OTP attendance, and travel constraints are frequently highlighted by patients as a reason for not continuing treatment (Beardsley et al., 2003) (Kim et al., 2024). Our findings corroborate other studies that have highlighted high barriers to accessing OTPs via public transportation (Kim et al., 2024; Ngo and Anand, 2024). This extended travel time on public transit can constitute a barrier to OUD treatment that may render it inaccessible, increasing opioid-related overdose risks.

Our results suggest notably lower access to OTPs on public transit in early morning hours, which is when many patients travel to receive methadone treatment (Frank, 2021) (Table 2). Increasing public transit service in the early morning, when transit service is typically less frequent (Hicks, 2017), could expand access to OTPs. Future research should examine ways to improve access to OTPs for patients without access to a car in the early morning. Patients may not be able to regularly travel to OTPs at later times during the day due to employment or familial obligations. If patients are unable to access OTPs via transit in the early-morning hours, they may forgo treatment, increasing risks for returning to illicit opioid use and opioid-related overdose.

For communities where travel times to an OTP using public transportation were longer than 30 min, non-medical emergent transit (NEMT) could improve access to OTPs. NEMT is transportation to a medical facility via a third-party provider in a car or American Disability Act (ADA)-accessible van. While Medicaid patients in Massachusetts (MassHealth) can access NEMT through PT-1 waivers depending on their coverage (MassHealth, 2025), additional funding could potentially expand this option to OTP patients on non-eligible insurance plans, including patients who are not enrolled in Medicaid. In addition, these communities may benefit from the expansion of access to methadone via a new OTP or a mobile clinic to mitigate the travel burden of treatment.

Prior research on disparities in healthcare access have shown that people of color experience larger transportation barriers when accessing care (Dumas, 2015; Shuman et al., 2024). In contrast, our study demonstrated that decedents in municipalities with a greater percentage of non-Hispanic White residents were less likely to have access to an OTP within 30 min by public transit in Greater Boston. However, this relationship was the opposite in the City of Boston, although not statistically significant. Notably, people of color constituted greater than one-sixth of the population in only 9 of 49 municipalities analyzed in Greater Boston, which includes the City of Boston, compared to 10 of the 24 neighborhoods in the City of Boston (Lima et al., 2025) (Census.gov, 2021). Moreover, OTPs are typically located in communities with more non-Hispanic Black and Hispanic residents due to NIMBY-ism, complex zoning regulations, and the lasting influence of the War on Drugs (Goedel et al., 2020; Horn et al., 2019; Jehan et al., 2024). Finally, many municipalities in Greater Boston are suburban and had lower transit frequency when compared to the City of Boston, based on GTFS data. Thus, our contrasting results in Greater Boston when compared to prior studies on transit-based access to healthcare are likely explained by the large number of predominantly non-Hispanic White suburbs in the area, which have poor OTP access via public transit due to lower transit frequency and lower odds of OTPs being placed in those communities. While this association is statistically significant, it may be subject to unmeasured confounding from, for example, an individual's socioeconomic status.

Despite the context of OTP placement and the counterintuitive trend of differences in access to OTPs based on race/ethnicity in Greater Boston, our results highlighted communities with high proportions of non-Hispanic Black and Hispanic residents that had poor access to OTPs in the City of Boston (Hyde Park, West Roxbury, Roslindale, and Dorchester) (Supplementary Table 3). In addition to transportation barriers, these communities are also more likely to be low-income and uninsured or underinsured (Wu et al., 2016), making them more susceptible to the impacts of OUD and less likely to be able to access treatment. Improving other facets of access to OTPs, such as lowering costs and offering culturally informed services is especially important in these communities to lower overdose risk.

Our study has several limitations. First, we only considered geographic access to OTPs, which is one of many barriers to accessing methadone treatment. We were unable to account for OTP capacity and waitlists, or a patient's insurance coverage and ability to attend an OTP while it is open. Healthcare providers may not have been able to provide culturally competent care to patients or treat underlying mental health conditions that often accompany addiction. Second, we did not know how many patients in Boston used public transportation to access methadone treatment. Future research should examine how patients are travelling to OTPs, for how long they travel, in addition to how receiving methadone interacts with their daily work commutes. Third, decedent residences are not a perfect proxy for OTP patients; the distribution of OTP patient residences may be different than that of decedents. While it would be more representative to use a sample of current OTP patients or people living with OUD, these data only exist as estimates and lack the spatial (i.e., address) component necessary for our study. Further, we were unable to include decedents without a residence at the time of their death, which may have excluded unhoused individuals. Fourth, our statistical models do not account for confounding variables that may influence the odds of a decedent's residence being within 30 min of an OTP on public transit, such as the decedent's socioeconomic status.

In addition, there are limitations based on our choice of GIS methods. First, we used the smallest of ten service areas generated during a 30-min period. Owen and Levinson (Owen and Levinson, 2015) used a similar methodology to calculate travel times to workplaces for a departure of every minute between 7 am and 9 am in the Twin Cities (Minnesota) area. Unlike our study, their analysis did not utilize the smallest service area. However, given the high prevalence of delays on the MBTA (Hicks, 2017), we believe that our choice to use the minimum is practical. This is also a unique strength of our study, which avoided unrealistic assumptions that a patient precisely coordinates their arrival at a stop with the bus or train, and that the bus or train runs perfectly on schedule. Accordingly, our results highlight areas with unmet OTP access needs based on a more realistic 30-min travel time on transit that does not arbitrarily inflate the time budget of an OTP patient.

Finally, we used ESRI's public transportation tool, which is unable to divide the total travel time into separate walk-time, wait-time, and transit-time. This means that service areas may have been modeled so that the patient walked for 30 min if no bus or train arrived during that time. It is unlikely that most patients would walk 30 min to an OTP.

## 5 Conclusions

We examined access to methadone treatment at OTPs in Boston via travel on public transit and walking to assess inequities in access to treatment and implications for opioid-related overdose risks. Our results highlight a need for increased transit frequency and alternative transportation options due to poor OTP access on public transit. We identified municipalities and neighborhoods with elevated unmet methadone treatment needs that require urgent attention to increase treatment access to help prevent opioid-related morbidity and mortality. We found mixed results on inequities by race and ethnicity. Our results can help inform targeted public health and clinical interventions to address unmet methadone treatment needs.

## Funding credits and disclosure of potential and real conflicts of interest

This research was supported by the Massachusetts Department of Public Health. Our MDPH Engagement Contract ID is INTF2400HH2500224557. All authors declare no conflicts of interest.

## CRediT authorship contribution statement

**Max R. O'Reilly:** Writing – review & editing, Writing – original draft, Visualization, Software, Methodology, Formal analysis, Conceptualization. **Thomas J. Stopka:** Writing – review & editing, Supervision, Project administration, Funding acquisition, Conceptualization. **Ric Bayly:** Writing – review & editing, Methodology, Conceptualization. **Olivia Lewis:** Writing – review & editing, Data curation, Conceptualization. **Shikhar Shrestha:** Writing – review & editing, Conceptualization. **Jack Cordes:** Writing – review & editing, Conceptualization. **Alexander Y. Walley:** Writing – review & editing, Conceptualization. **Sumeeta Srinivasan:** Writing – review & editing, Supervision, Methodology, Conceptualization.

## Funding

This work was supported by the Massachusetts Department of Public Health. Our MDPH engagement ID is INTF2400HH2500224557.

## Declaration of competing interest

The authors declare that they have no known competing financial interests or personal relationships that could have appeared to influence the work reported in this paper.

## Data Availability

The authors do not have permission to share data.

## Appendix A Supplementary data

Supplementary table 1 Demographic information on fatal opioid-related overdose decedents in Greater Boston and the City of Boston 2020-2022. Supplementary table 1

Supplementary table 2 Percent of fatal opioid-related decedent residences within 30 minutes of an OTP by public transportation in each town receiving MBTA service by race and ethnicity, Greater Boston Area, 2020-2022. Supplementary table 2

Supplementary table 3 Percent of fatal opioid-related decedent residences within 30 minutes of an OTP by public transportation in each neighborhood of the City of Boston by race and ethnicity, 2020-2022 Supplementary table 3
