# Supplementary material for: Public transit and methadone – Spatial analyses of opioid treatment program access in Greater Boston, 2020–2022
Source: Prev Med Rep. 2025 Nov 20;60:103317. doi: 10.1016/j.pmedr.2025.103317 (PMC12681875; doi:10.1016/j.pmedr.2025.103317)
Supplement: Supplementary table 1 — Demographic information on fatal opioid-related overdose decedents in Greater Boston and the City of Boston 2020-2022. [file mmc2.docx]

**Supplementary Table 1 –** demographic information on fatal opioid-related overdose decedents in Greater Boston and the City of Boston 2020-2022.

|  | *Greater Boston* | *City of Boston* |
| --- | --- | --- |
| *Total Number of Decedents* | 2073 | 711 |
| *Proportion Male* | 72.8% | 74.3% |
| *Proportion >30* | 88.1% | 89.9% |
| *Proportion non-Hispanic White* | 65.4% | 40.5% |
| *Proportion non-Hispanic Black* | 18.3% | 34.2% |
| *Proportion Hispanic* | 14.7% | 23.1% |
| *Proportion Other Race/Ethnicity* | 1.6% | 2.1% |
| *Proportion Single* | 70.0% | 70.1% |
