# Supplementary material for: Public transit and methadone – Spatial analyses of opioid treatment program access in Greater Boston, 2020–2022
Source: Prev Med Rep. 2025 Nov 20;60:103317. doi: 10.1016/j.pmedr.2025.103317 (PMC12681875; doi:10.1016/j.pmedr.2025.103317)
Supplement: Supplementary table 2 — Percent of fatal opioid-related decedent residences within 30 minutes of an OTP by public transportation in each town receiving MBTA service by race and ethnicity, Greater Boston Area, 2020-2022. [file mmc3.docx]

**Supplementary Table 2**. Percent of fatal opioid-related decedent residences within 30 minutes of an OTP by public transportation in each town receiving MBTA service by race and ethnicity, Greater Boston Area, 2020-2022.

| Town Name | Percent Access; all decedents | Percent access; non-Hispanic white decedents | Percent access; non-Hispanic Black decedents | Percent access; Hispanic decedents | Percent access; other or race ethnicity |
| --- | --- | --- | --- | --- | --- |
| TOTAL | 50.1% | 41.0% | 69.1% | 67.7% | 45.5% |
| Arlington | 62.5% | 57.1% | 100.0% | - | 100.0% |
| Avon | 0.0% | 0.0% | 0.0% | - | - |
| Bedford | 0.0% | 0.0% | 0.0% | - | 0.0% |
| Belmont | 0.0% | 0.0% | 0.0% | - | - |
| Beverly | 0.0% | 0.0% | 0.0% | - | - |
| Boston | 80.5% | 81.7% | 79.1% | 81.8% | 66.7% |
| Braintree | 31.0% | 36.0% | 0.0% | - | 0.0% |
| Brookline | 71.4% | 80.0% | 100.0% | 0.0% | - |
| Burlington | 0.0% | 0.0% | - | - | - |
| Cambridge | 92.9% | 91.7% | 100.0% | 75.0% | - |
| Canton | 0.0% | 0.0% | - | 0.0% | - |
| Chelsea | 97.4% | 100.0% | 100.0% | 92.9% | - |
| Danvers | 5.9% | 6.3% | - | 0.0% | - |
| Dedham | 0.0% | 0.0% | 0.0% | 0.0% | 0.0% |
| Everett | 14.3% | 13.2% | 33.3% | 12.5% | - |
| Hingham | 0.0% | 0.0% | 0.0% | - | - |
| Holbrook | 0.0% | 0.0% | 0.0% | - | - |
| Hull | 0.0% | 0.0% | - | - | - |
| Lexington | 0.0% | 0.0% | - | - | 0.0% |
| Lincoln | 0.0% | 0.0% | - | - | - |
| Lynn | 70.4% | 65.0% | 81.3% | 75.0% | 75.0% |
| Malden | 8.6% | 8.9% | 0.0% | 20.0% | - |
| Marblehead | 0.0% | 0.0% | - | 0.0% | - |
| Medford | 51.3% | 48.6% | 66.7% | 100.0% | - |
| Melrose | 0.0% | 0.0% | 0.0% | 0.0% | - |
| Milton | 33.3% | 33.3% | - | - | - |
| Nahant | 0.0% | 0.0% | - | - | - |
| Needham | 0.0% | 0.0% | - | - | - |
| Newton | 7.7% | 8.7% | 0.0% | - | 0.0% |
| Norwood | 0.0% | 0.0% | - | 0.0% | - |
| Peabody | 11.9% | 16.1% | 0.0% | 0.0% | 0.0% |
| Quincy | 31.7% | 32.4% | 30.8% | 30.0% | 0.0% |
| Randolph | 0.0% | 0.0% | 0.0% | 0.0% | - |
| Reading | 12.5% | 12.5% | - | - | - |
| Revere | 66.2% | 62.0% | 83.3% | 77.8% | - |
| Salem | 0.0% | 0.0% | 0.0% | 0.0% | - |
| Saugus | 16.1% | 17.2% | 0.0% | 0.0% | - |
| Somerville | 100.0% | 100.0% | 100.0% | 100.0% | 100.0% |
| Stoneham | 9.5% | 5.3% | - | 50.0% | - |
| Swampscott | 0.0% | 0.0% | 0.0% | - | - |
| Wakefield | 0.0% | 0.0% | 0.0% | - | - |
| Walpole | 0.0% | 0.0% | 0.0% | 0.0% | - |
| Waltham | 10.5% | 6.9% | 0.0% | 40.0% | 0.0% |
| Watertown | 0.0% | 0.0% | 0.0% | 0.0% | - |
| Westwood | 0.0% | 0.0% | - | - | - |
| Weymouth | 46.2% | 46.0% | 100.0% | 0.0% | - |
| Winchester | 0.0% | 0.0% | - | - | - |
| Winthrop | 0.0% | 0.0% | 0.0% | 0.0% | - |
| Woburn | 13.5% | 12.9% | 0.0% | 50.0% | 0.0% |
