# Supplementary material for: Public transit and methadone – Spatial analyses of opioid treatment program access in Greater Boston, 2020–2022
Source: Prev Med Rep. 2025 Nov 20;60:103317. doi: 10.1016/j.pmedr.2025.103317 (PMC12681875; doi:10.1016/j.pmedr.2025.103317)
Supplement: Supplementary table 3 — Percent of fatal opioid-related decedent residences within 30 minutes of an OTP by public transportation in each neighborhood of the City of Boston by race and ethnicity, 2020-2022 [file mmc4.docx]

**Supplementary Table 3.** Percent of fatal opioid-related decedent residences within 30 minutes of an OTP by public transportation in each neighborhood of the City of Boston by race and ethnicity, 2020-2022

| Neighbor-hood in the City of Boston | Percent Access; all decedents | Percent Access; non-Hispanic White decedents | Percent Access; non-Hispanic Black decedents | Percent Access; Hispanic decedents | Percent Access; other race or ethnicity decedents |
| --- | --- | --- | --- | --- | --- |
| Total | 80.4% | 81.6% | 79.0% | 81.7% | 66.7% |
| Allston | 100.0% | 100.0% | - | 100.0% | 100.0% |
| Back Bay | 100.0% | 100.0% | 100.0% | 100.0% | - |
| Beacon Hill | 100.0% | 100.0% | 100.0% | 100.0% | 100.0% |
| Brighton | 100.0% | 100.0% | 100.0% | 100.0% | 100.0% |
| Charlestown | 100.0% | 100.0% | - | 100.0% | - |
| Chinatown | 100.0% | 100.0% | 100.0% | 0.0% | - |
| Dorchester | 62.4% | 57.1% | 67.0% | 63.6% | 20.0% |
| Downtown | 100.0% | 100.0% | 100.0% | 100.0% | - |
| East Boston | 92.5% | 90.0% | 75.0% | 100.0% | 100.0% |
| Fenway | 100.0% | 100.0% | 100.0% | 0.0% | - |
| Hyde Park | 6.3% | 0.0% | 7.1% | 9.1% | - |
| Jamaica Plain | 95.7% | 87.5% | 100.0% | 100.0% | 100.0% |
| Longwood | 100.0% | 100.0% | - | 0.0% | - |
| Mattapan | 96.9% | 100.0% | 94.4% | 100.0% | 100.0% |
| Mission Hill | 80.0% | 50.0% | 100.0% | 100.0% | 100.0% |
| North End | 100.0% | 100.0% | - | 0.0% | - |
| Roslindale | 57.9% | 62.5% | 33.3% | 71.4% | 0.0% |
| Roxbury | 97.6% | 100.0% | 94.8% | 100.0% | - |
| South Boston | 100.0% | 100.0% | 100.0% | 100.0% | - |
| South Boston Waterfront | 100.0% | 100.0% | - | 0.0% | - |
| South End | 100.0% | 100.0% | 100.0% | 100.0% | 100.0% |
| West End | 100.0% | 100.0% | 100.0% | 0.0% | - |
| West Roxbury | 4.8% | 5.9% | 0.0% | 0.0% | - |
